# Uniportal Video-Assisted Thoracoscopic Surgery Completion Lobectomy Long after Wedge Resection or Segmentectomy in the Same Lobe: A Bicenter Study

**DOI:** 10.3390/cancers16071286

**Published:** 2024-03-26

**Authors:** Elisa Meacci, Majed Refai, Dania Nachira, Michele Salati, Khrystyna Kuzmych, Diomira Tabacco, Edoardo Zanfrini, Giuseppe Calabrese, Antonio Giulio Napolitano, Maria Teresa Congedo, Marco Chiappetta, Leonardo Petracca-Ciavarella, Carolina Sassorossi, Marco Andolfi, Francesco Xiumè, Michela Tiberi, Gian Marco Guiducci, Maria Letizia Vita, Alberto Roncon, Anna Chiara Nanto, Stefano Margaritora

**Affiliations:** 1 Department of General Thoracic Surgery, Fondazione Policlinico Gemelli IRCCS, Catholic University of Sacred Heart of Rome, 00168 Rome, Italy; khrystyna.kuzmych01@icatt.it (K.K.); diomira_tabacco@yahoo.it (D.T.); giuseppe93calabrese@virgilio.it (G.C.); antoniogiulionapolitano@gmail.com (A.G.N.); mariateresa.congedo@policlinicogemelli.it (M.T.C.); marco.chiappetta@policlinicogemelli.it (M.C.); leonardo.petraccaciavarella@policlinicogemelli.it (L.P.-C.); sassorossi.caro@gmail.com (C.S.); marialetizia.vita@policlinicogemelli.it (M.L.V.); stefano.margaritora@unicatt.it (S.M.); 2 Department of Thoracic Surgery, Ospedali Riuniti, 60126 Ancona, Italy; majed.refai@ospedaliriuniti.marche.it (M.R.); michele.salati@ospedaliriuniti.marche.it (M.S.); marco.andolfi@ospedaliriuniti.marche.it (M.A.); francesco.xiume@ospedaliriuniti.marche.it (F.X.); michela.tiberi@ospedaliriuniti.marche.it (M.T.); gianmarco.guiducci@ospedaliriuniti.marche.it (G.M.G.); alberto.roncon@ospedaliriuniti.marche.it (A.R.); annachiara.nanto@ospedaliriuniti.marche.it (A.C.N.); 3 Service of Thoracic Surgery, University Hospital of Lausanne, 1005 Lausanne, Switzerland; edoardo.zanfrini@chuv.ch

**Keywords:** uniportal VATS, single port, completion lobectomy, completion pneumonectomy, segmentectomy, NSCLC

## Abstract

**Simple Summary:**

Completion lobectomy (CL) entails the resection of the remaining pulmonary lobe subsequent to wedge resection or segmentectomy. Indications for CL include reoperations for multiple or relapsed lung cancers and metastatic lung tumors, and the prognostic advantage of these procedures has been widely reported. However, ipsilateral surgical treatments, particularly within the same lobe, present challenges due to the development of intrapleural adhesions, rendering reoperation more difficult and time-consuming. VATS has emerged as the gold standard in the surgical treatment of early-stage NSCLC, offering superior postoperative outcomes when compared to thoracotomy. Its efficacy has been well established, even during complex procedures. However, its application in ipsilateral reoperations remains anecdotal, and to the best of our knowledge, no studies have analyzed the safety and efficacy of uniportal-VATS in this setting. This paper aims to evaluate the role of iniportal-VATS in CL long after wedge resection or anatomical segmentectomy in the same lobe.

**Abstract:**

Background: Completion lobectomy (CL) following a prior resection in the same lobe may be complicated by severe pleural or hilar adhesions. The role of uniportal video-assisted thoracoscopic surgery (U-VATS) has never been evaluated in this setting. Methods: Data were collected from two Italian centers. Between 2015 and 2022, 122 patients (60 men and 62 women, median age 67.7 ± 8.913) underwent U-VATS CL at least 4 weeks after previous lung surgery. Results: Twenty-eight (22.9%) patients were affected by chronic obstructive pulmonary disease (COPD) and twenty-five (20.4%) were active smokers. Among the cohort, the initial surgery was performed using U-VATS in 103 (84.4%) patients, triportal-VATS in 8 (6.6%), and thoracotomy in 11 (9.0%). Anatomical segmentectomy was the initial surgery in 46 (37.7%) patients, while hilar lymphadenectomy was performed in 16 (13.1%) cases. CL was performed on 110 (90.2%) patients, segmentectomy on 10 (8.2%), and completion pneumonectomy on 2 (1.6%). Upon reoperation, moderate pleural adhesions were observed in 38 (31.1%) patients, with 2 (1.6%) exhibiting strong adhesions. Moderate hilar adhesions were found in 18 (14.8%) patients and strong adhesions in 11 (9.0%). The median operative time was 203.93 ± 74.4 min. In four (3.3%) patients, PA taping was performed. One patient experienced intraoperative bleeding that did not require conversion to thoracotomy. Conversion to thoracotomy was necessary in three (2.5%) patients. The median postoperative drainage stay and postoperative hospital stay were 5.67 ± 4.44 and 5.52 ± 2.66 days, respectively. Postoperative complications occurred in 34 (27.9%) patients. Thirty-day mortality was null. Histology was the only factor found to negatively influence intraoperative outcomes (*p* = 0.000). Factors identified as negatively impacting postoperative outcomes at univariate analyses were male sex (*p* = 0.003), age > 60 years (*p* = 0.003), COPD (*p* = 0.014), previous thoracotomy (*p* = 0.000), previous S2 segmentectomy (*p* = 0.001), previous S8 segmentectomy (*p* = 0.008), and interval between operations > 5 weeks (*p*= 0.005). In multivariate analysis, only COPD confirmed its role as an independent risk factor for postoperative complications (HR: 5.12, 95% CI (1.07–24.50), *p* = 0.04). Conclusions: U-VATS CL seems feasible and safe after wedge resection and anatomical segmentectomy.

## 1. Introduction

Completion lobectomy (CL) involves resection of the remaining pulmonary lobe subsequent to wedge resection or segmentectomy. Indications for CL primarily include reoperations for metachronous or relapsed non-small cell lung cancer (NSCLC) and metastatic lung tumors, and the prognostic advantages of these procedures has been widely reported [1,2].

Since the majority of CLs are performed several weeks after previous surgery, the development of dense parietal and hilar adhesion after initial resection can make the recognition and isolation of anatomical structures extremely difficult. The contorted anatomical structures and fibroplastic proliferation in the thoracic apex, chest wall, and, particularly, between pulmonary vessels and bronchial stumps pose a high risk of pulmonary artery injury during repeated surgery [3,4,5]. The limited operative field and weakened haptic feedback during thoracoscopic operations have historically discouraged surgeons from performing ipsilateral re-operative major lung resection by VATS, especially when CL is required after a previous anatomical segmentectomy [6,7,8]. This is because the bronchovascular components have already been denuded and manipulated during the previous segmentectomy. Despite the successful application of VATS, particularly U-VATS, in many challenging situations such as sleeve and double sleeve lobectomies, pneumonectomy, and even tracheal and carinal plastic surgery [9,10,11,12], ipsilateral reoperation remains a prohibited area. Safe management of difficult hila has become one of the greatest challenges, even for thoracotomy [13].

Nevertheless, due to the advantages offered by VATS when compared to open surgery, such as smaller incision, reduced postoperative pain, shorter hospital stays, less inflammatory response, faster recovery, and higher patient satisfaction [14], the National Comprehensive Cancer Network has recommended the VATS approach as the preferred surgical approach for early-stage non-small cell lung cancer (NSCLC). Therefore, it is important to establish the feasibility and safety of VATS for CL.

Recently, several studies have described successful CL procedures, [4,8,15,16,17,18,19,20,21,22,23,24,25,26,27] without increasing postoperative complications [17,18]. However, only a few studies, which evaluated a small number of patients or isolated case reports, have explored the role of VATS reoperations [4,8,19,20,21]. Furthermore, no studies have focused on the adoption of uniportal VATS for CL. As of now, it remains unknown whether this challenging procedure affects patient safety and recovery.

In this study, we aimed to retrospectively investigate the experience of two Italian centers with extensive expertise in uniportal VATS, focusing on patients undergoing uniportal VATS CL after previous ipsilateral wedge resection or anatomical segmentectomy in the same lobe.

CL following diagnostic wedge resection using video-assisted thoracoscopic surgery (VATS) may be feasible and safely performed [15,28], as described in many papers. Despite the potential risk of errhysis or major bleeding associated with the dissection of intrapleural adhesions, the procedure has been described as viable.

Although cases of CL after anatomical segmentectomy have been reported [8,16,19,29], performing CL after segmentectomy in the same lobe can be complicated by severe adhesions around the hilar structures, especially around the pulmonary artery (PA) and lung parenchyma [4,5]. The distortion of hilar structures following the initial operation, particularly scar formation in the pulmonary trunks, can make the recognition and isolation of anatomical structures extremely difficult [6,7,8]. This is because the bronchovascular components have already been denuded and manipulated during the previous segmentectomy. Studies have reported that CL may become more difficult if a long period has elapsed since the previous segmentectomy [4,8]. Fatal bleeding and lung injury may also occur. Specifically, CL after segmentectomy in the same lobe is a challenging procedure that requires adhesiolysis of dense hilum tissue. Only a few studies have reported the surgical outcomes of CL long after segmentectomy in the same lobe [4,19,20,21,22].

## 2. Materials and Methods

Clinical data of 122 patients who underwent U-VATS CL at least 4 weeks after previous lung surgery in the same lobe, between 2015 and 2022, were collected among two Italian Thoracic Centers with wide experience in U-VATS (Thoracic Surgery Unit at Fondazione Policlinico UniversitarioA.Gemelli-Rome and Thoracic Surgery Unit Azienda Ospedaliero-Universitaria delle Marche).

The study was approved by our IRB and conducted in accordance with the ethical standards of the Declaration of Helsinki and its later amendments. Individual informed consent was waived due to the retrospective nature of the study and the anonymity of patients enrolled.

The main clinical and surgical variables reviewed were:

(1) Clinical: sex, age, comorbidities (COPD, diabetes, hypertension, cardiovascular, other disease), smoking history, history of previous neoplasm, pulmonary function (FEV1%, FVC%, TIFF, DLCO), ASA score, ECOG score.
(2) First surgical operation: side, lobe, access (open, uniportal VATS, multiportal VATS), number of resections, resection type (wedge or anatomical segmentectomy), lymph-node harvested (stations), histology.
(3) Second surgical operation: interval between operations, degree of parietal adherences, degree of hilar adherences, surgical procedure in reoperation (lobectomy or pneumonectomy), operative time, blood loss, PA taping, intraoperative complications, postoperative complications, conversion to thoracotomy, drainage stay, postoperative stay, air leakage, bleeding, pneumonia, embolism, 30-day mortality.

Each clinical case underwent thorough discussion by internal multidisciplinary teams, comprising radiologists, oncologists, and thoracic surgeons, to approve the indication for lung reoperation. Indications for CL included radical resection in patients with a history of prior cancer where the lesion was primarily expected to be metastatic but final histopathology revealed an unexpected primary lung cancer, cancer relapse in the same lobe after sublobar resection, and reiterative surgery of metastases in the same lobe, encompassing all visible tumor lesions in patients with only lung metastases.

Before undergoing CL, all patients were evaluated by routine blood tests, electrocardiography (or other cardiologic second level tests, if necessary), pulmonary function test, and total-body computed tomography (CT). U-VATS CL was performed by highly skilled surgeons proficient in U-VATS technique.

### 2.1. Surgical Procedure

**Figure 1:**
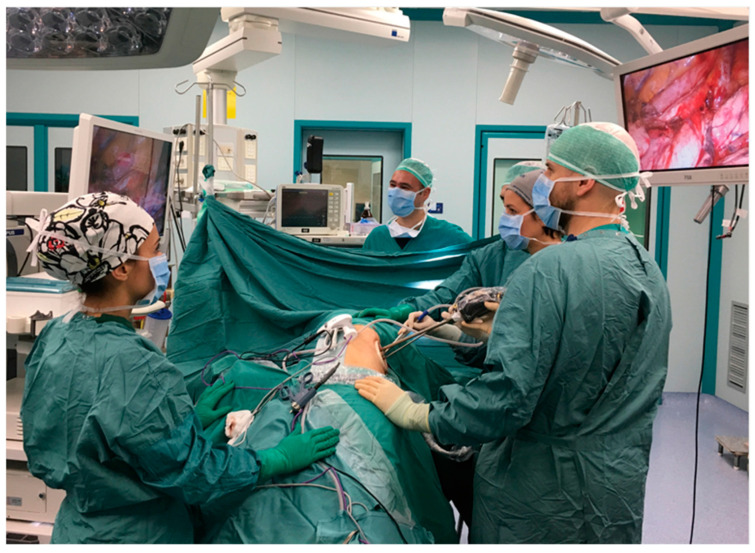
Patient’s position.

CL was performed under general anesthesia and double-lumen intubation. Patients were positioned in lateral decubitus (Figure 1) with arms flexed and stretched towards their head. A single 3–4 cm muscle-sparing incision was made on the midaxillary line in the IV or V intercostal space, depending on the location of the lesion. For lesions localized in the upper lobes or centrally, the IV space was preferred. A wound protector was placed, and a 10 mm 30° thoracoscope and endoscopic instruments were introduced through the same incision, with the camera positioned in the upper part of the incision. The width of the incision combined with the good mobilization of the lung achievable with the curved shaped, dual pivot instruments allowed a good palpation of lung parenchyma for localizing nodules. Endostaplers and energy dissectors were used for dissecting and cutting lung parenchyma and vascular structures, following the same oncological radicality principles observed in open surgery. A complete lymphadenectomy was performed in case of primary lung cancer.

The specimen was removed using an Endobag. At the end of each procedure, an extrapleural paravertebral intercostal nerve block was performed by infiltrating 3 mL of ropivacaine (4.75 mg/mL) in 3–4 intercostal spaces above and below the incision, under endoscopic view. Usually, only one chest drain tube (24 or 28 Fr) was placed at the end of the operation through the same incision and in its upper part.

#### Postoperative Management

All patients received effective thoracic analgesia, including local nerve block during the operation and systemic administration of painkillers. Additionally, patients were instructed to engage in early mobilization and undergo respiratory physiotherapy in the immediate postoperative period.

A chest X-ray was performed after the operation and the day prior to the expected chest tube removal. Chest tubes were removed once no evidence of air leaks was detected and the drainage volume fell below 200–250 mL within a 24-h period.

### 2.2. Statistical Analysis

All categorical variables were reported as absolute numbers and percentages (%), while continuous variables were expressed as mean followed by standard deviation. Kolmogorov–Smirnov test was used to evaluate normal distribution of data. Categorical variables were compared by Chi-squared test, while continuous variables by independent sample Student’s *t*-test if normal distributed or by Mann–Whitney U-test if not normal.

Clinical-pathological and surgical variables were tested in univariate analysis to assess factors associated with risk of conversion, intraoperative and postoperative complications. All covariates with *p* < 0.2 were selected for the Cox proportional hazards regression model to assess independent risk factors.

A *p*-value less than 0.05 was considered statistically significant. Statistical analysis was performed using IBM SPSS Statistics for Macintosh, Version 25.00 (Armonk, NY, USA).

## 3. Results

A total of 122 patients (60 (49.2%) men, median age 67.7 ± 8.91 years) who underwent U-VATS CL were enrolled in the study.

General information on the patients is summarized in Table 1.

Twenty-eight (22.9%) patients were affected by chronic obstructive pulmonary disease (COPD) and twenty-five (20.4%) were active smokers. Among the cohort, the initial surgery was performed using U-VATS in 103 (84.4%) patients, triportal-VATS in 8 (6.6%), and thoracotomy in 11 (9.0%). Fifty-nine (48.4%) resections were performed on the right side. Anatomical segmentectomy was the initial surgery in 46 (37.7%) patients, while hilar lymphadenectomy was performed in 16 (13.1%) cases. CL was performed on 110 (90.2%) patients, while 10 (8.2%) underwent completion segmentectomy and 2 (1.6%) completion pneumonectomy. At reoperation, moderate pleural adhesions were observed in 38 (31.1%) patients, and 2 (1.6%) patients had strong ones. Moderate hilar adhesions were found in 18 (14.8%) patients, and 11(9.0%) presented strong adhesions, as seen in Table 2. The median operative time was 203.93 ± 74.4 min. PA taping was performed in four (3.3%) patients. Intraoperative bleeding occurred in one patient but did not require conversion to thoracotomy. Conversion to thoracotomy was necessary in three (2.5%) patients (two due to pleural adhesions and one due to hilar adhesions). The median lymph-node harvest was 4.94 + 4.416. The median postoperative drainage stay was 5.67 + 4.44 days. Forty-three (35.2%) patients experienced postoperative complications, including 25 cases of air leak, 6 of bleeding, 6 of pneumonia, and 6 of atrial fibrillation. No reoperation was required. The median postoperative stay was 5.52 + 2.66 days. Thirty-day mortality was null (Table 3).

In univariate analysis, the only factor negatively influencing intraoperative outcomes was histology (*p* = 0.000). Smoking history was associated with a higher risk of conversion (*p* = 0.048), while previous surgical approach (thoracotomy) was shown to positively influence the development of parietal adhesions (*p* = 0.013).

Factors negatively impacting postoperative outcomes at univariate analyses were male sex (*p* = 0.003), age > 60 years (*p* = 0.003), COPD (*p* = 0.014), previous thoracotomy (*p* = 0.000), previous S2 segmentectomy (*p* = 0.001), previous S8 segmentectomy (*p* = 0.008), and interval between operations > 5 weeks (*p*= 0.005), as seen in Table 4. According to three different time intervals (5 weeks, >5 and <15 weeks, >15 weeks), we found no differences in terms of operative time (*p* = 0.27), blood loss (*p* = 0.68), and p.o. drainage stay (*p* = 0.123).

We found a significant difference (*p* = 0.015) in terms of postoperative hospital stay, in particular evaluating the difference in terms of median p.o stay between the Group <5 weeks (3.75 ± 1.39 days) vs. Group >15 weeks (6.80 ± 4.02 days) (Table 5).

In multivariate analysis, only COPD confirmed its role as an independent risk factor for postoperative complications (HR: 5.12, 95% CI (1.07–24.50), *p* = 0.04).

## 4. Discussion

Reoperations for multiple lung cancers have been extensively performed for NSCLC and metastatic lung tumors, and their prognostic advantage has been widely reported [1,2]. However, ipsilateral reoperations, particularly in the same lobe, pose challenges due to the development of intrapleural adhesions, making the reoperation more difficult and time requiring. Despite VATS represents the gold standard in the surgical treatment of early-stage NSCLC, and its role has been well established even during complex procedures [9,10,11,12], its adoption in ipsilateral reoperation remains anecdotal. To our knowledge, no studies have analyzed the safety and efficacy of uniportal-VATS (U-VATS) in this setting.

In this paper, we report the surgical outcomes of patients who underwent U-VATS CL in the same lobe long after wedge resection or anatomical segmentectomy.

Our findings indicate that U-VATS CL was not associated with high risk of intraoperative and postoperative complications. No perioperative mortality was reported in our experience. This is noteworthy, considering that mortality rates for patients who have undergone repeated pulmonary resection in the past, particularly via open techniques, have been reported to range from 5 to 11% [23,30,31,32,33]. The dramatic reduction in mortality and complication rates for reiterative lung surgery may be attributable to recent improvements in surgical procedures and technology. Additionally, more recently, experiences evaluating the outcome of reiterative VATS or open surgery have reported no mortality and very low rates of complications during reoperation (Table 6).

Several authors have evaluated the efficacy and safety of both VATS and open ipsilateral reoperations [4,8,17,21,25], indicating that CL after diagnostic wedge resection using multiportal-VATS may be feasible and safely performed [15,28].

However, a only few studies have focused on surgical outcomes of CL after anatomical segmentectomy [4,8,19,20,21,22]. This is primary due to the complexity of CL after segmentectomy in the same lobe, which can be complicated by severe adhesions around the hilar structures, particularly around the pulmonary artery (PA) and lung parenchyma [4,5]. This often necessitate meticulous adhesiolysis of dense hilum tissue.

The timing of pleural adhesion formation postoperatively has not been investigated. However, considering pleural adhesions as a type of wound healing, as classified by Moore [34,35], it has been suggested that a rough estimate of 5 weeks can be used to anticipate the development of more severe adhesions.

In our experience, a time interval of more than 5 weeks between the previous surgery and CL was found to be statistically significant (*p* < 0.005) in univariate analysis regarding the relationship between perioperative factors and postoperative complications.

Other studies have also reported that CL may become more difficult with a longer interval since the previous segmentectomy [4,8], thus confirming our data. For instance, LiU [25] compared the impact of adhesions on surgical outcomes using an eight-week interval to CL. Their results aligned with Omasa’s [8] findings, where longer operative times and more severe adhesions were observed in the long interval to CL group (56–1470 days vs. 3–35 days). Moreover, a tendency for increased operative bleeding (*p* = 0.055), greater usage of fibrin glue (*p* = 0.080), and significantly longer operative time (*p* = 0.036) were noted in the long interval to CL group. Additionally, injury to the pulmonary arteries was experienced only in the long interval to CL group (3/6 cases).

Takahashi [4] described CLs performed at least a month after the previous lung cancer segmentectomies and found that hilum adhesions were particularly severe after superior mediastinal nodal dissection during the previous lung cancer segmentectomies. In our experience, the diagnosis of NSCLC at first operation was associated with an increased intraoperative complication rate (*p* < 0.000), and this result is probably associated with the lymphadenectomy that we performed during the first operation.

In our study, the incidence of postoperative complications was negatively influenced (*p* < 0.000) by using thoracotomy in first surgery (11/122 patients 9%). This result is confirmed by Takahashi [4], who first compared patients undergoing VATS or thoracotomy CL. They found that more severe adhesions (72% vs. 42%, *p* = 0.06) occurred in the thoracotomy group compared to the VATS group, although the difference was not statistically significant. Undoubtedly, the presence of dense parietal and hilar adhesions complicates the surgical procedure. Dissecting of parietal adhesions can be challenging, often complicated by lesion on the visceral pleura, which may lead to postoperative air leaks despite corrective intraoperative maneuvers.

Postoperative complications observed in the present study were mostly represented by air leakage (20.5%), followed by bleeding, pneumonia, and arrhythmia.

Similarly, as in our series, the most common complication of secondary pulmonary resection is shown to be air leakage [26,27].

Conversely, the development of severe hilar adhesion subsequent to a prior segmentectomy underscores the importance of securing the main PA before starting hilar dissection to prevent catastrophic bleeding, especially when it is challenging to expose and divide the PA. However, even though it is often recommended, only 4 (9%) patient received PA taping (4 /46 undergoing CL after segmentectomy) in our experience, 1/5 in Takahashi’s [4] and 2/12 in Liu’s [21] experience. Despite this low percentage of PA taping, PA injury during VATS-CL was negligible in our series and 20% (1/5 patients) in the series by Takahashi [4]. Paradoxically, the incidence of PA injury is higher during open surgery CL, as reported by Omasa [8] (3/6–50%), probably due to the preoperative selection of patients. During the VATS procedure, it is also difficult to isolate the main pulmonary artery in patients with adhesion around the origin. Therefore, at our institute, we adequately expose the pulmonary artery to facilitate clamping it with forceps in the cases of severe hilum adhesion, and PA taping is performed when that pulmonary artery is completely isolated.

Our operation time of 209.93 ± 74.40 min showed to be consistent with the results previously reported. Takahashi [4], when comparing VATS vs. open thoracotomy for CL after segmentectomy, found no differences between the two techniques (VATS and open, respectively, 259 vs. 339 min *p* = 0.55) [M], as in Takamori’s [20] (138–234 vs. 165–407 min) and Liu’s experiences (272 vs. 253 min) [21]. The long time spent to perform ipsilateral reoperations does not appear to be associated with operation-related mortality or a significant increase in postoperative complications. In our study, we observed 34 (27.9%) postoperative complications, predominantly persistent air leaks (25 cases (73.5% of whole post-operative complications)) among 110 CL (46 after anatomical segmentectomy on the same lobe), 10 completion segmentectomies, and 2 completion pneumonectomies. Motono, [18], in their analysis of 41 patients who underwent repeated pulmonary resections by VATS or thoracotomy, found that the postoperative complication rate of the first operation was 29%, and that of the second operation was again 29%. No mortality was registered in his global experience. Holbeck [15], in 2016, was the first to systematically assess the safety of ipsilateral multiportal-VATS CL after diagnostic wedge resection by comparing it with standard VATS lobectomy (SL). They found no differences between groups in terms of major or minor complications, and 30-day mortality rate was 0% vs. 1.1% for the CL group and the SL group, respectively (*p* = 0.99).

Similar results were reported by Sun [27], who investigated the feasibility of multiportal-VATS in ipsilateral reoperation for major lung resection (lobectomy or segmentectomy, excluding pneumonectomy). Among 14 patients analyzed, 9 patients underwent lobectomy and 5 underwent segmentectomy during the second operation.

Postoperative drainage stay in our series was 5.67 ± 4.44 days, and postoperative hospital stay was 5.52 ± 2.66 days. Other authors analyzing patients who underwent CL after segmentectomy by multiportal-VATS reported similar results: postoperative drainage stay was 3 days (2–7) in Takahashi’s experience [4], 6.7 ± 4.2 days in Sun’s [27] experience, and 1 day in Takamori’s [20] one.

Postoperative stay ranged between 6 and 14 days in the similar literature [4,20,22,25,27].

Conversion to thoracotomy was necessary in three patients (2.5%) in our series. Conversions from complete VATS to open surgery have been described in a few studies, with an incidence ranging from 6.5 to 23% [36,37].

In our multivariable logistic regression analysis on postoperative complications, we found that COPD might be a predictor of postoperative complications (HR: 5.12, 95% CI (1.07–24.50), *p* = 0.04), mainly represented by air leak. Therefore, based on our results in this integrated analysis, patients affected by COPD undergoing CL are more likely to exhibit persistent air leak.

## 5. Conclusions

The increasing adoption of segmentectomy in the treatment of NSCLC, driven by the growing number of early lung cancer cases and the increased incidence of second primary lung cancer or local recurrence, has led to a rise in repeated ipsilateral thoracic operations after primary lung cancer surgery. This highlights the necessity, particularly in the era of minimally invasive techniques, to effectively perform CL or re-segmentectomy after a previous segmentectomy on the ipsilateral side and the same lobe. Given the advantages of U-VATS compared to open surgery, it is crucial to establish the role of this minimally invasive approach in such a setting.

In conclusion, the results of this study suggest that secondary U-VATS pulmonary resection is both feasible and safe for patients undergoing ipsilateral CL, not only following wedge resection but also, and predominantly, after segmentectomy. Adhesions should no longer be considered a contraindication to U-VATS reoperations. To our knowledge, this study represents the first collection and analysis of data on uniportal-VATS CL. Nonetheless, the retrospective nature and dual-center setting of this investigation inevitably introduce selection bias. The high volume of operations in our hospitals and the extensive experience of the surgeons performing CLs may also have contributed to the lower incidence of intra and postoperative complications observed. We expect more prospective multicenter studies to confirm our results.

## Figures and Tables

**Table 1:** Characteristics of patients who underwent U-VATS CL.

Characteristics	N (122)	%
Age (years)	67.7 ± 8913	
Sex (male)	60	49.20%
Active smoker	25	20.4
Pack/year	35.35 ± 20.5	
Former smoker	53	43.4
Previous neoplasms	64	52.4
COPD	28	22.9
Diabetes	10	8.1
Hypertension	62	50.8
Cardiovascular	49	40.2
Other diseases	57	46.7
FEV1%	86.30 ± 22.86	
FVC%	95.55 ± 18.80	
TIFF	5.70 ± 18.75	
DLCO	69.29 ± 23.97	
ASA	1.93 ± 0.706	
ECOG	1.35 ± 0.513	

**Table 2:** Characteristics of previous surgery.

Characteristics	N (122)	%
Previous operation side (right/left)	59/63	48.4/51.6
Site of previous resection (RUL/ML/RLL; LUL/LLL)	34/9/16; 27/36	27.8/7.3/13.1; 22.1/29.4
Number of previous resections (each surgery)	1.07 ± 2.262	
Harvested lymph nodes	4.94 ± 4.41	
Previous access (uniportal/multiportal/open)	103/8/11	84.4/6.6/9
Site of previous anatomical segmentectomy	46 * (S1:8/S2:4/S3:4/S5:2/S6:9/S7:7/S8:5/S9:1/S10:5/S11:1)	6.6/3.3/3.3/1.6/7.4/5.7/4.1/0.8/4.1/0.8.
Previous diagnosis (NSCLC/MLC/O)	109/5/8	89.3/4/6.6

* Summary of harvested segments; RUL right upper lobe, ML middle lobe, RLL right lower lobe, LUL left upper lobe, LLL left lower lobe; NSCLC non-small cell lung cancer, MLC metastatic lung cancer, O other.

**Table 3:** Outcomes of completion lobectomy.

Outcomes	N (122)	%
Procedure of second operation (L/S/P)	110/10/2	90/8.2/1.8
Site of second operation (RUL/RML/RLL; LUL/LLL)	34/9/16; 27/36	27.8/7.3/13.1; 22.1/29.4
Degree of parietal adherences	1.08 ± 0.818	
Degree of hilar adherences	0.64 ± 0.773	
Operative time	209.93 ± 74.40	
Estimated blood loss (mL)	250 ± 312	
PA taping	4 (post segmentectomy)	8.6
Conversion to thoracotomy	3	2.5
Bleeding	1	0.8
Severe hilar adhesions	1	0.8
Severe parietal adhesions	1	0.8
Postoperative Complications	34	27.9
Air leak	25	20.4
Bleeding	6	4.9
Pneumonia	6	4.9
AF	6	4.9
Postoperative drainage stay (days)	5.67 ± 4.44	
Postoperative stay (days)	5.52 + 2.66	
30-day mortality	0	0

L lobectomy, S segmentectomy, P pneumonectomy.

**Table 4:** Univariate analysis on postoperative complications.

Variable	*p*-Value
**Sex (M)**	**0.003**
**Age > 60 y**	**0.003**
Smoking	0.628
Cardiovascular disease	0.58
**COPD**	**0.014**
NSCLC as first diagnosis	0.364
**Previous thoracotomy**	**0.000**
Side	0.822
**Segment S2**	**0.001**
Segment S8	**0.008**
CL site	0.381
Previous lymph-node dissection	0.767
**Time interval > 5 weeks**	**0.005**

**Table 5:** Univariate analysis on intraoperative complications.

Variable	*p*-Value
Sex	0.303
Age	0.112
Smoking	0.608
Cardiovascular disease	0.216
COPD	0.580
**NSCLC as first diagnosis**	**0.000**
Previous thoracotomy	0.910
Side	0.335
Segment	0.100
CL site	0.405
Time interval > 5 weeks	1.000

**Table 6:** Literature review.

	Approach (VATS/Open)	Adhesions/Hilar Fibrosis	Degree of Adhesions (None, Mild, Severe)	Operative Time (min)	PA Taping (Yes/No)	Securing of Main PA (Yes/No)	Compilations (Yes/No)	Conversion	Blood Loss (mL)	Drainage Duration (Days)	Mean H Stay (Days)	Mortality
Omasa (2016) [8]	(V) 11 (T) 0	8 (73)	3/0/8	216 ± 89	5 (45%)	5/0	6 (54.5)	N/A	300 ± 314	5.1 ± 3.4	N/A	0
Holbek (2016) [15]	(V) 80 (T) 0	65 (81)	N/A	110 (95–140)	N/A	N/A	7 (8.75)	1 (1.3%)	100 (50–238)	2 (1–5)	4 (2–6)	0
Chen (2018) [17]	(V) 36 (T) 28	28 (78) 22 (79)	8/0/28 6/0/22	3.7 ± 1.0 h 3.4 ± 0.9 h	N/A	N/A	N/A	0 -	354 ± 211.6 432.1 ± 396.1	5.7 ± 4.0 7.1 ± 6.1	11.0 ± 5.4 20.4 ± 9.5	0 0
Takahashi (2019) [4]	(V) 5 (T) 5	5 (100) 5 (100)	0/2/3 0/1/4	259 (279–389) 339 (201–458)	1 2	N/A	2 (40) 3 (60)	1 -	350 (200–950) 500 (160–6870)	3 (2–7) 2 (1–7)	9–14 8–18	0
Liu (2019) [21]	(V) 1 -	1	0/0/1	350	0	0	0	0	450	N/A	3	0
Sun (2020) [27]	(V) 14 -	10 (71)	0/9/1	2.2 ± 0.5 h	0	0	0	1 (7%)	203.6 ± 126.3	6.7 ± 4.2	5.9 ± 4.6	0
Suzuki (2021) [19]	- (T) 4	4 (100)	0/3/1	64–164	1	1	1 (25)	0	75–370	N/A	7–21	0
Motono (2021) [18]	(V) 36 (T) 4	N/A	N/A	126 (46–501)	N/A	N/A	8 (29)	N/A	N/A	N/A	12 (4–27)	0
Komatsu (2021) [22]	(V) 1 -	1 (100)	0/1/0	266	0	0	0	0	200	N/A	13	0
Chen (2023) [26]	(V) 70 -	68 (97)	2/11/29	120 (30–472)	N/A	N/A	17 (24)	10	50 (3–600)	N/A	6 (2–16)	0
Takamori (2021) [20]	(V) 3 (T) 5	3 (100) 5 (100)	0/2/1 0/1/4	138–234 165–407	0 2	1 3	1 (100) 1 (100)	0 -	61–253 230–2194	1 1–6	5–6 5–10	0 0
Liu (2022) [25]	(V) 12 (T) 29	5 (42) 21 (72)	0/0/5	272 (198–317) 253 (199–317)	2 (17%) 10 (34%)	N/A	3 (25) 14 (48)	0 -	229 (160–410) 381 (200–432)	N/A	8 (6–12) 9 (7–13)	0 0

## Data Availability

The data presented in this study are available in this article and can be shared up on request.
